# Proteomic and metabolomic profiling reveals the fungicidal mechanisms of *Microsporum canis* in response to methylene blue-mediated photodynamic therapy

**DOI:** 10.3389/fmicb.2025.1734090

**Published:** 2026-01-12

**Authors:** Gaoyuan Peng, Shulei Qin, Weilun Xu, Cunwei Cao, Kaisu Pan, Lan Huang, Liuwei Liao, Junmeng Zhou, Dongyan Zheng, Xinyu Zhang

**Affiliations:** 1 Department of Dermatology and Venereology, The First Affiliated Hospital of Guangxi Medical University, Nanning, China; 2 Guangxi Key Laboratory of Mycosis Research and Prevention, Nanning, China; 3 Fangchenggang Wanqing Institute of Mycosis Prevention and Control, Fangchenggang, China

**Keywords:** antifungal mechanism, metabolomics, *Microsporum canis*, photodynamic therapy, proteomics

## Abstract

**Background:**

*Microsporum canis* is a primary causative agent of dermatophytosis. Its rising antifungal resistance necessitates the development of effective therapeutic alternatives. Although methylene blue-mediated photodynamic therapy (MB-PDT) is a promising strategy, a system-level understanding of its fungicidal mechanism is lacking.

**Methods:**

An integrated multi-omics approach was employed, using data-independent acquisition (DIA) proteomics and untargeted metabolomics, to map the molecular response of clinical *M. canis* isolates to MB-PDT. Pathway enrichment analysis was performed to elucidate the key biological processes affected.

**Results:**

MB-PDT induced multi-faceted molecular perturbations in *M. canis*. The treatment simultaneously disrupted membrane integrity by downregulating ergosterol biosynthesis (e.g., C4-methylsterol oxidase) and impaired the fungus’s antioxidant defenses by suppressing key enzymes such as glutathione S-transferase. Critically, the treatment suppressed secreted virulence factors essential for host invasion, including subtilisin-like protease 7. These disruptions led to a profound suppression of core biosynthetic machinery, with ribosome biogenesis and translation identified as the most significantly inhibited pathways. This resulted in a collapse of protein synthesis, energy production, and amino acid metabolism.

**Conclusion:**

The results indicate that the efficacy of MB-PDT stems from a multi-target mechanism that simultaneously damages cellular structures, attenuates virulence, and dismantles the fungus’s metabolic and translational capacity. This contrasts sharply with single-target conventional antifungals, providing a strong molecular rationale for its low potential to induce resistance. This study offers a comprehensive molecular blueprint for the action of MB-PDT against *M. canis*, strongly supporting its development as a durable therapeutic strategy for dermatophytosis.

## 1 Introduction

*Microsporum canis*, a primary zoophilic dermatophyte, is a leading cause of cutaneous infections such as *tinea corporis* and *tinea capitis*, particularly in children (Pasquetti et al., 2017). The management of these infections is increasingly complicated by the global rise of antifungal resistance. This trend compromises the efficacy of standard therapies, leading to a significant clinical challenge characterized by treatment failures and recurrent disease (Dellière et al., 2024; Martinez-Rossi et al., 2018). Recent studies indicate that terbinafine resistance rates have reached up to 25% in certain populations as of 2024, exacerbating this issue (Gupta et al., 2025). This growing crisis underscores the urgent need for alternative therapeutic strategies that can effectively eradicate resistant fungal pathogens (Gold et al., 2024; McCormick and Ghannoum, 2024).

In this context, methylene blue-mediated photodynamic therapy (MB-PDT) has emerged as a compelling alternative antifungal strategy (Cabral et al., 2021; Scwingel et al., 2012). This approach utilizes methylene blue (MB), a non-toxic photosensitizer that generates cytotoxic reactive oxygen species (ROS) upon activation by a specific wavelength of light (Chen et al., 2019). The selection of MB is particularly advantageous due to its established clinical safety record, cost-effectiveness, and high ROS generation efficiency, making it an ideal candidate for topical applications (Baltazar et al., 2015). Unlike conventional antifungals that act on specific molecular targets, the indiscriminate oxidative damage induced by MB-PDT affects multiple cellular components simultaneously, thereby reducing the likelihood of resistance development (Gupta et al., 2023).

While the fungicidal efficacy of MB-PDT is well-documented, a comprehensive understanding of the molecular mechanisms underlying its action against *M. canis* remains to be fully elucidated (Voit et al., 2021; Liang et al., 2016). The systemic proteomic and metabolic reprogramming that leads to cell death is not fully understood (Corrêa et al., 2025; Lam et al., 2011). The present study addresses this critical knowledge gap by employing an integrated proteomic and metabolomic approach (Xie et al., 2025; Culibrk et al., 2016; Yang et al., 2019). This combined strategy was specifically applied to cross-validate molecular perturbations across key biological processes, including membrane lipid metabolism, protein biosynthesis, and redox homeostasis. By using clinical isolates of *M. canis*, the translational relevance of the findings is enhanced of our findings and provide a system-level perspective on the fungus’s response to MB-PDT (Marbaniang et al., 2025; Moskaluk et al., 2022).

This study aims to systematically elucidate the multi-pronged fungicidal mechanisms of MB-PDT, moving beyond phenotypic observations to map the specific pathways disrupted by the treatment. Elucidating these complex interactions holds significant translational potential. The insights gained can provide a foundation for optimizing clinical PDT protocols, identifying novel biomarkers for treatment efficacy, and designing rational combination therapies to combat recalcitrant dermatophyte infections.

## 2 Materials and methods

**Figure 1:**
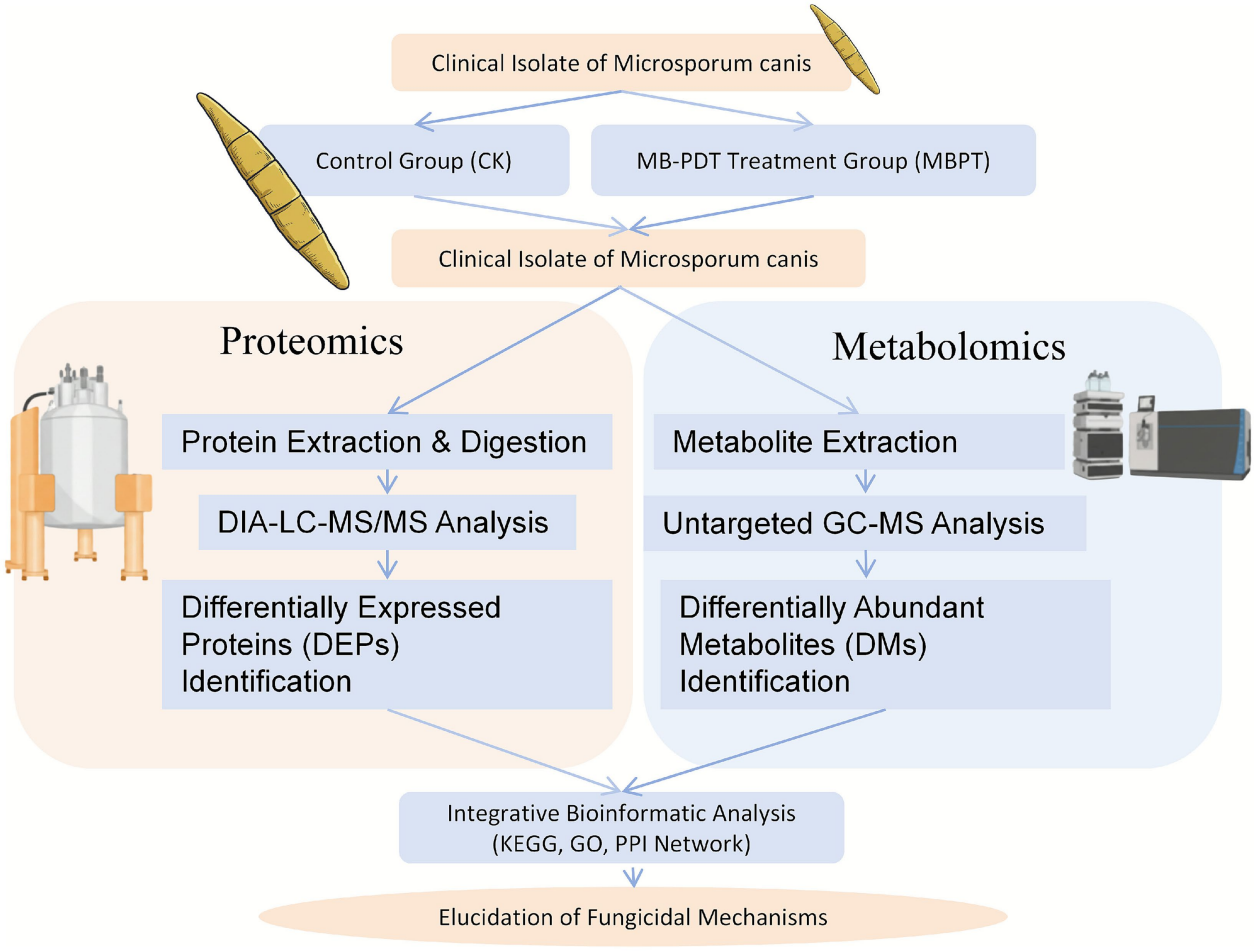
Schematic overview of the experimental workflow. Clinical isolates of *Microsporum canis* were divided into a control group (CK) and an MB-PDT treatment group (MBPT). Samples from both groups were subjected to parallel proteomic and metabolomic analyses. The resulting multi-omics data were integrated using bioinformatic tools to elucidate the fungicidal mechanisms of MB-PDT.

The overall experimental workflow is schematically illustrated in Figure 1. This study employed an integrated proteomic and metabolomic approach to investigate the molecular response of clinical *Microsporum canis* isolates to MB-PDT. Samples were divided into a control group (CK) and an MB-PDT treatment group (MBPT), followed by parallel proteomic and metabolomic analyses to identify differentially expressed proteins (DEPs) and differentially abundant metabolites (DMs). The resulting data were then integrated through bioinformatic analysis to elucidate the fungicidal mechanisms.

### 2.1 Fungal strains, cultivation, and identification

The *Microsporum canis* strains used for this study (NYQ, HJN, and A2939) were clinical isolates obtained from skin specimens of patients at the Dermatology Outpatient Department of the First Affiliated Hospital of Guangxi Medical University. Strain identification was confirmed using a combination of morphological and molecular methods. Morphological analysis included microscopic examination of mycelia and spores. For molecular confirmation, the internal transcribed spacer (ITS) region of each strain was amplified and sequenced, and the resulting sequences were aligned against public databases to verify species identity. The strains were cultured in a fungal incubator (SPX-250B-Z, Shanghai Boxun Industrial Co., Ltd., Shanghai, China) at 28 °C for 10 days. A fungal suspension was then prepared and adjusted to a final concentration of 1 × 10^6^ CFU/mL. All experiments were conducted using three independent biological replicates for each strain and condition.

### 2.2 Methylene blue-mediated photodynamic therapy

A sub-inhibitory concentration of MB-PDT was used to induce a cellular stress response while ensuring fungal viability for subsequent proteomic and metabolomic analysis. To determine this concentration, the minimum inhibitory concentration (MIC) was established using a broth microdilution method, defined as the lowest concentration of methylene blue (MB) that inhibited visible fungal growth following irradiation with a 632 ± 10 nm red light LED source (PL-D, Peninsula Medical, Chongqing, China) at an energy density of 80 J/cm^2^. The average MIC under these conditions was found to be 0.5 μg/mL. For the omics experiments, fungal cultures were incubated with methylene blue (MB; Macklin, Shanghai, China), prepared from a freshly diluted 1% stock solution, at a final concentration of 0.25 μg/mL (1/2 MIC) for 2 h in the dark, followed by irradiation for 20 min at an energy density of 80 J/cm^2^. The three biological replicates subjected to this procedure were designated as the treatment groups (MBPT; samples NYQ2, HJN2, and A29392). Parallel cultures that received no treatment (neither MB nor light) were designated as the control groups (CK; samples NYQ1, HJN1, and A29391). Following the treatment procedure, all cultures were incubated for an additional 24 h at 28 °C before being harvested.

### 2.3 Sample collection for omics analysis

At the conclusion of the 24-h incubation period, fungal cells from both the MBPT and CK groups were harvested by centrifugation at 5,000 × g for 10 min at 4 °C. The cell pellets were washed twice with ice-cold PBS (pH 7.2) to remove residual media. The final pellets were immediately flash-frozen in liquid nitrogen and stored at −80 °C until proteomic and metabolomic profiling.

### 2.4 Proteomic profiling

For proteomic analysis, fungal pellets were homogenized in SDS lysis buffer (4% SDS, 100 mM Tris–HCl, pH 7.6) with a protease inhibitor cocktail using an ultrasonic cell disruptor. The lysate was clarified by centrifugation, and the supernatant was collected. Protein concentration was determined using a BCA Protein Assay Kit (P0012, Beyotime, China), and protein quality was verified by 12% SDS-PAGE with Coomassie blue staining. A 100 μg protein aliquot from each sample was reduced with 100 mM DTT at 95 °C for 5 min and alkylated with 100 mM iodoacetamide (IAM) in darkness for 30 min. Proteins were then processed by filter-aided sample preparation (FASP) and digested with trypsin (Promega, United States) at a 1:50 enzyme-to-protein ratio for 20 h at 37 °C. The resulting peptides were desalted using a peptide desalting spin column (Thermo Fisher Scientific, United States) and analyzed on a Vanquish Neo UHPLC system connected to an Orbitrap Astral mass spectrometer (Thermo Fisher Scientific, United States). Peptide separation was performed on an EASY-Spray^™^ C18 column (150 μm × 15 cm, 2 μm; ES906) at 55 °C using a gradient of mobile phase A (0.1% formic acid in water) and B (0.1% formic acid in 80% acetonitrile). The mass spectrometer was operated in data-independent acquisition (DIA) mode, with full MS scans acquired at 240,000 resolution over a 380–980 *m*/*z* range. MS/MS analysis utilized 299 overlapping isolation windows of 2 *m*/*z* with an HCD collision energy of 25 eV.

The raw DIA files were analyzed using DIA-NN software (v1.8.1) against the *Microsporum canis* protein database (GCF_000151145.1). Search parameters included trypsin cleavage (max one missed cleavage), carbamidomethylation of cysteine (fixed modification), and oxidation of methionine and N-terminal acetylation (variable modifications), with a false discovery rate (FDR) of ≤1%. Data was pre-processed by filtering for proteins identified in at least 50% of samples within a group, and missing values were imputed using the *k*-nearest neighbors (KNN) algorithm. The mass spectrometry proteomics data have been deposited to the ProteomeXchange Consortium via the PRIDE partner repository with the dataset identifier PXD069640.

### 2.5 Metabolomic profiling

For metabolomic analysis, fungal pellets were homogenized in a pre-chilled 4:1 (v/v) methanol–water solution (methanol; Sinopharm Chemical Reagent Co., Ltd., Shanghai, China) to extract and quench metabolites. After sonication and centrifugation, the supernatant was collected and dried. Metabolite profiling was conducted on a UPLC system coupled to a high-resolution mass spectrometer (Thermo Fisher Scientific, United States), using a C18 reversed-phase column for separation. The mass spectrometer operated in both positive and negative ion modes, acquiring data in full scan mode over a mass range of 70–1,050 *m*/*z*. Pooled quality control (QC) samples were injected at regular intervals to monitor system stability.

Raw data files were converted to .mzXML format using ProteoWizard and processed in R (v4.1.2) with the XCMS package for peak detection, retention time correction, and alignment. Metabolites were identified by matching their accurate *m*/*z* values (<10 ppm mass error) and MS/MS fragmentation patterns against public (HMDB, Metlin) and a proprietary in-house database (Shanghai Personalbio Technology Co., Ltd.). All annotations were classified as MSI Level 2 or higher. The metabolomics data have been deposited in the MetaboLights repository with the identifier MTBLS13188.

### 2.6 Bioinformatic and integrative omics analysis

Differentially expressed proteins (DEPs) were defined as those with a fold change >1.5 and an adjusted *p*-value <0.05. *p*-values were adjusted for multiple testing using the Benjamini–Hochberg procedure to control the FDR. Differentially abundant metabolites (DMs) were identified using a variable influence on projection (VIP) score >1.0 from the OPLS-DA model and a *p*-value <0.05. Both DEPs and DMs underwent Gene Ontology (GO) and Kyoto Encyclopedia of Genes and Genomes (KEGG) pathway enrichment analysis using the clusterProfiler package (V4.6.0). Protein–protein interaction (PPI) networks were generated with the STRING database (v12.0). For integrative analysis, both DEPs and DMs were mapped to KEGG pathways to identify commonly affected biological processes.

### 2.7 Statistical analysis

All statistical analyses were conducted in R (v4.1.2). Direct comparisons between the MBPT and CK groups were performed using a two-tailed Student’s *t*-test, for which a *p*-value <0.05 was considered statistically significant. Multivariate analyses, including principal component analysis (PCA) and orthogonal partial least squares discriminant analysis (OPLS-DA), were performed using the ropls package (V1.22.0). OPLS-DA models were validated through 7-fold cross-validation and permutation tests (*n* = 200) to ensure they were not overfitted.

## 3 Results

### 3.1 Proteomic profile of *Microsporum canis* in response to MB-PDT

#### 3.1.1 Identification and profile of differentially expressed proteins

**Figure 2:**
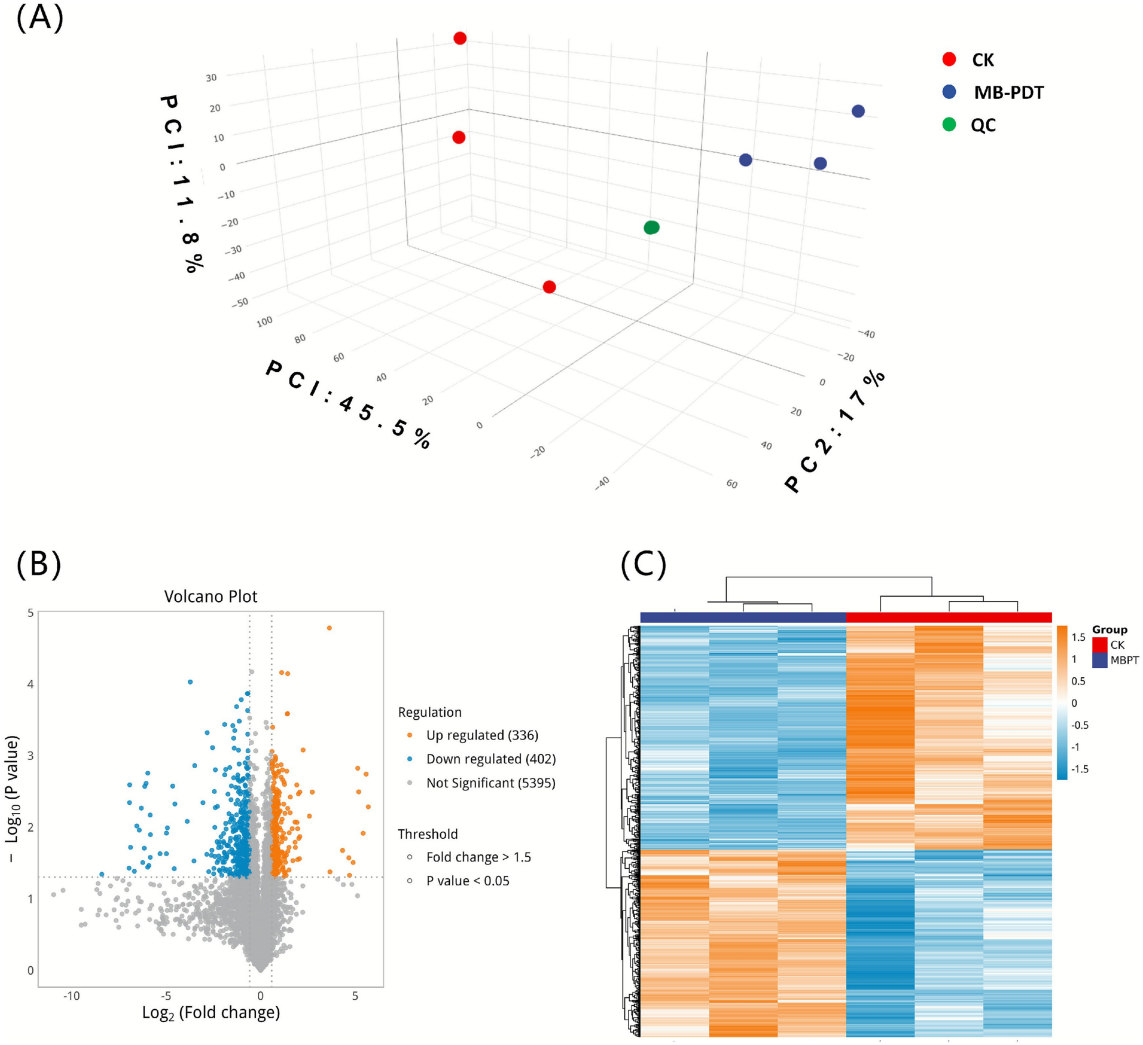
Global proteomic profile of *Microsporum canis* in response to MB-PDT. **(A)** 3D PCA score plot illustrating distinct proteomic profiles for MB-PDT exposed (MBPT, red), control (CK, blue), and quality control (QC, green) samples. The axes represent the top three principal components with their respective variance contributions (PC1: 45.5%, PC2: 17.0%, PC3: 11.8%). **(B)** Volcano plot of DEPs. Dashed lines indicate significance thresholds (fold change >1.5 and *p* < 0.05), highlighting 336 upregulated (orange) and 402 downregulated (blue) proteins. **(C)** Hierarchical clustering heatmap of the 738 DEPs. The color scale represents the *Z*-score normalized abundance of each protein (row) per sample (column).

Exposure to MB-PDT induced statistically significant and reproducible proteomic alterations in *M. canis*. Principal component analysis (PCA) confirmed that the proteomic profiles of the MB-PDT-exposed (MBPT) and control (CK) groups were distinctly separated (Figure 2A). Based on a fold change >1.5 and *p* < 0.05, 738 differentially expressed proteins (DEPs) were identified, comprising 336 upregulated and 402 downregulated proteins (Figures 2B,C). The overall proteomic response was characterized by a predominant downregulation of proteins. These DEPs are involved in key biological processes, including cell membrane integrity, virulence, and oxidative stress defense.

#### 3.1.2 Functional and pathway enrichment analysis of DEPs

**Figure 3:**
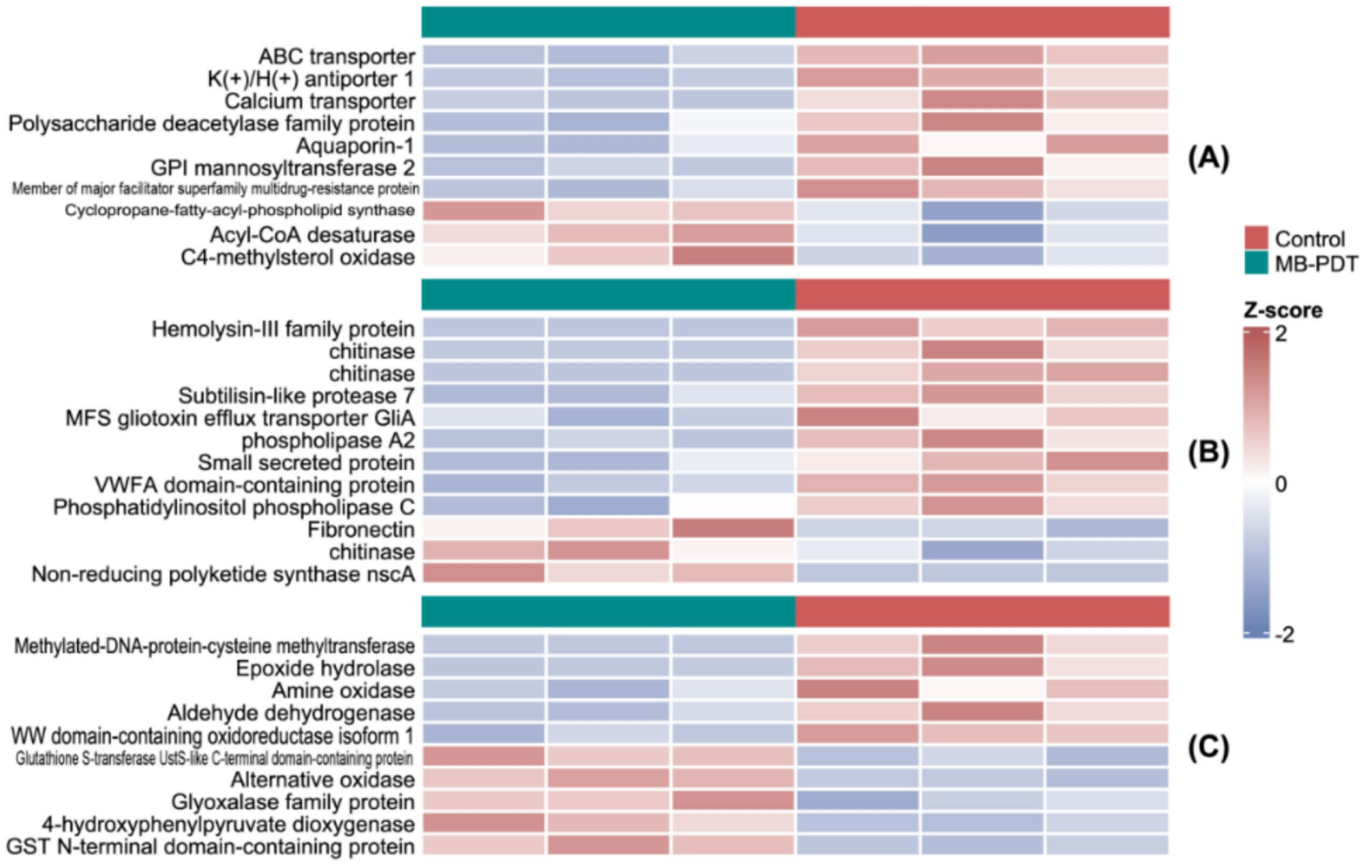
Heatmaps of key DEPs functionally categorized. The color indicates *Z*-score normalized expression (red: higher, blue: lower). Proteins are grouped by function. **(A)** Lipid metabolism and membrane transport. **(B)** Virulence factors. **(C)** Antioxidant and metabolic enzymes.

The identified DEPs were categorized based on their biological roles (Figure 3). A widespread downregulation was observed among proteins essential for cell membrane function (e.g., ABC transporter, K(+)/H(+) antiporter 1, acyl-CoA desaturase), virulence (e.g., multiple forms of chitinase, subtilisin-like protease 7, phospholipase A2, hemolysin-III family protein), and redox homeostasis (e.g., glutathione S-transferase, alternative oxidase, epoxide hydrolase) (Figures 3A–C).

**Figure 4:**
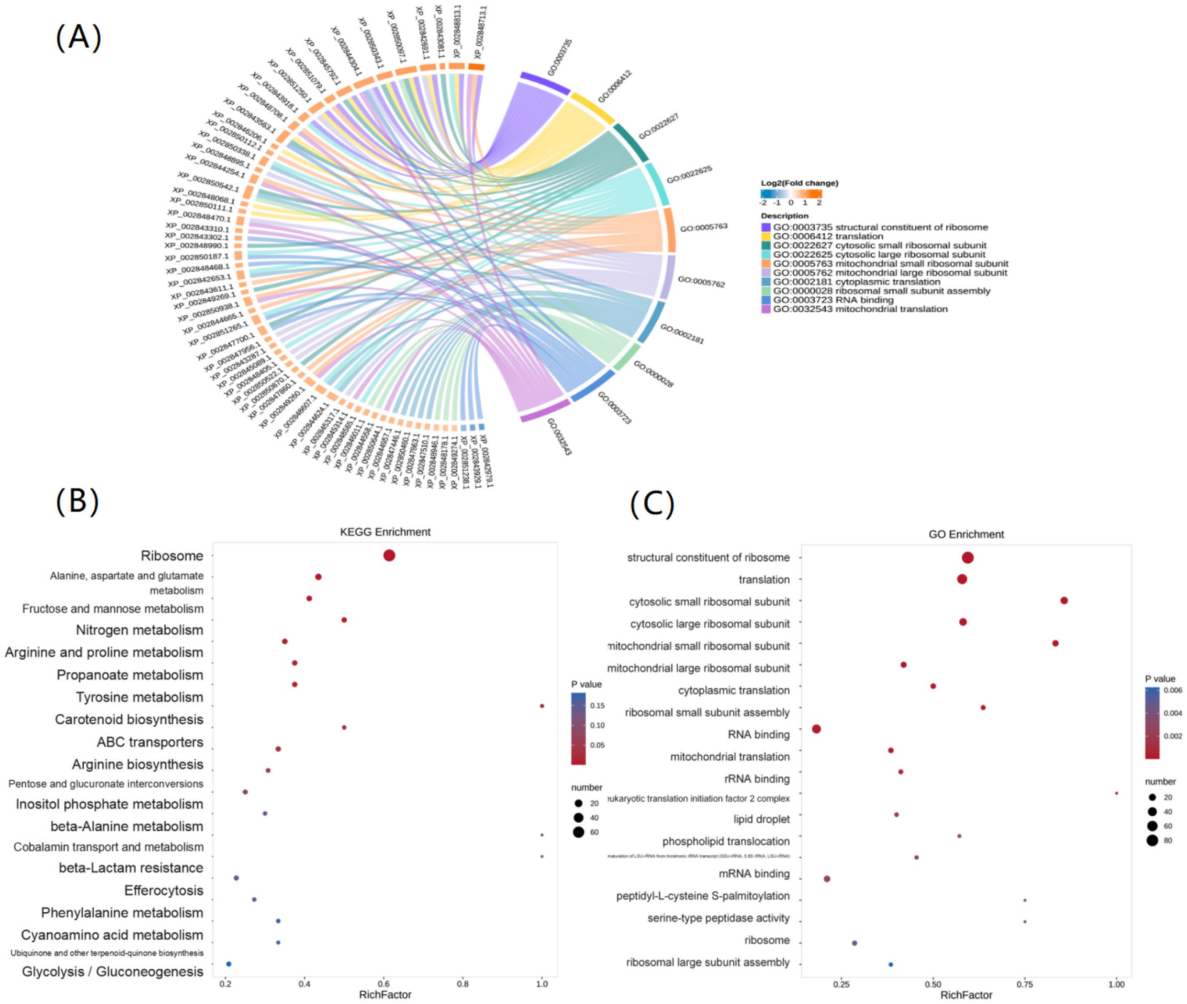
GO and KEGG pathway enrichment analysis of DEPs. **(A)** Chord diagram illustrating the relationship between enriched GO terms and their associated DEPs, with connecting bands colored by Log_2_(Fold change). **(B)** KEGG pathway enrichment bubble plot. The rich factor (*x*-axis) indicates enrichment level. Bubble size corresponds to the number of DEPs, and the color represents the *p*-value. **(C)** GO term enrichment plot; the axes and bubble properties are analogous to **(B)**.

Pathway enrichment analysis provided a systematic overview of these changes. Gene Ontology (GO) analysis indicated that DEPs were most significantly enriched in ribosome-related functions, including “structural constituent of ribosome” and “translation” (Figure 4C). Correspondingly, KEGG analysis identified “Ribosome” as the most significantly enriched pathway, followed by amino acid metabolism pathways such as “Alanine, aspartate and glutamate metabolism” and “Arginine and proline metabolism” (Figure 4B). The majority of proteins associated with these fundamental pathways were downregulated (Figure 4A).

### 3.2 Metabolomic profile of *Microsporum canis* in response to MB-PDT

**Figure 5:**
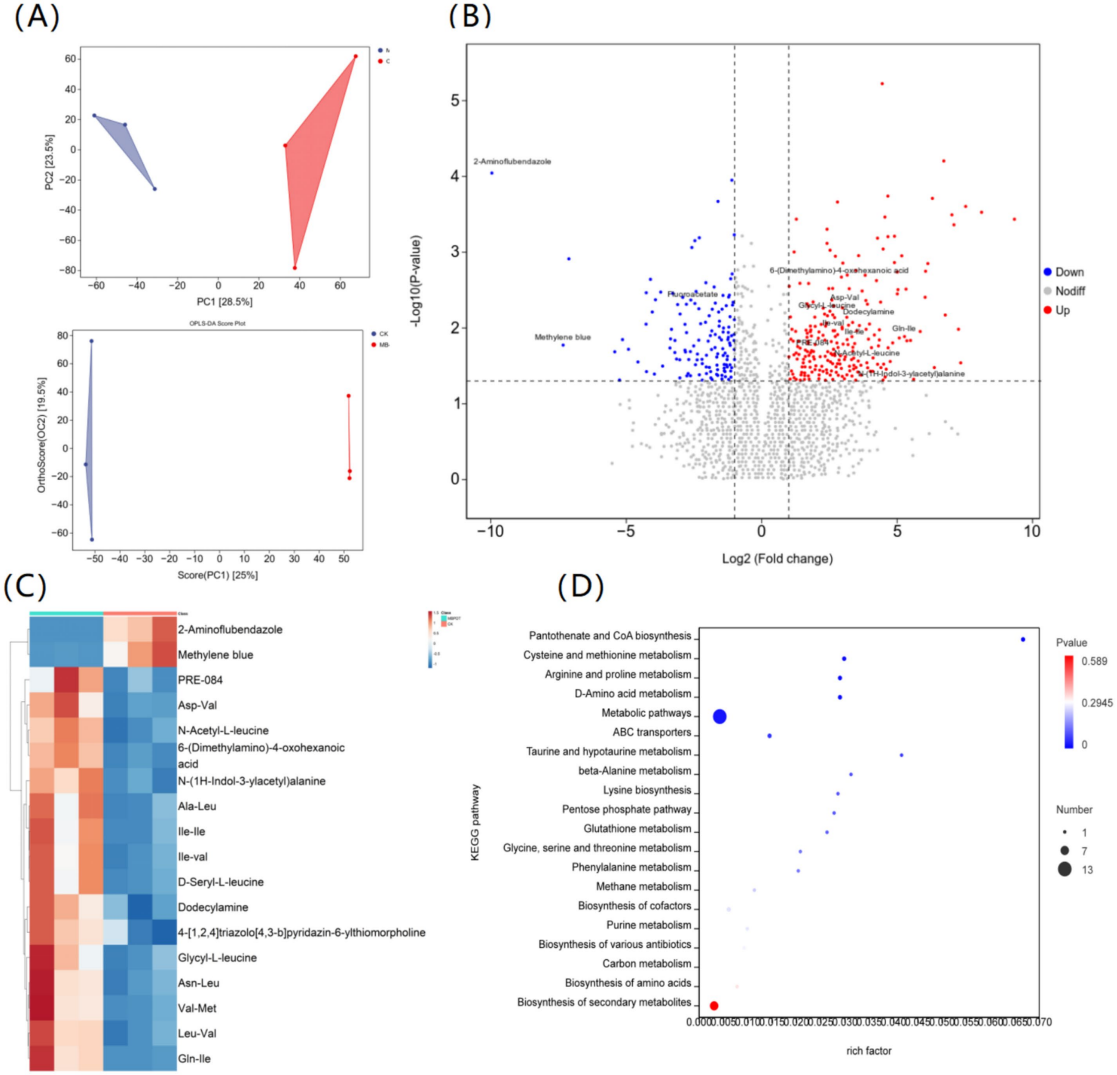
Metabolomic analysis of *M. canis* in response to methylene blue-mediated photodynamic therapy (MB-PDT). **(A)** Principal component analysis (PCA) and orthogonal partial least squares discriminant analysis (OPLS-DA) score plots showing distinct separation between MB-PDT (red) and control (CK, blue) groups. **(B)** Volcano plot depicting upregulated (red) and downregulated (blue) differentially abundant metabolites (DMs) based on *p*-value and fold change. **(C)** Heatmap of the most significantly altered DMs showing their relative abundances. **(D)** Kyoto Encyclopedia of Genes and Genomes (KEGG) pathway enrichment bubble plot for DMs. The rich factor (*x*-axis) indicates enrichment level, bubble size represents the number of DMs, and the color indicates the *p*-value.

To complement the proteomic findings, an untargeted metabolomic analysis was performed to profile metabolite changes. PCA and OPLS-DA confirmed a distinct metabolic separation between the MB-PDT and control groups (Figure 5A). Numerous DMs were identified (*p* < 0.05), revealing significant metabolic reprogramming (Figure 5B).

A substantial downregulation was observed for compounds such as 2-Aminoflubendazole (Log₂(Fold change) ≈ −9.5) and methylene blue (Log₂(Fold change) ≈ −4.8). Conversely, a prominent accumulation of various dipeptides was observed, including Asp-Val, Ala-Leu, Ile-Val, and Gln-Ile (Log₂(Fold change) values from +3 to +5), alongside other upregulated molecules such as N-(1H-Indol-3-ylacetyl)alanine (Log₂(Fold change) ≈ +6.2) (Figure 5C).

KEGG pathway analysis of DMs showed significant enrichment in “Metabolic pathways,” “ABC transporters,” and various amino acid metabolism pathways, including “Arginine and proline metabolism,” “D-Amino acid metabolism,” and “Cysteine and methionine metabolism” (Figure 5D). This metabolic profile was consistent with the observed alterations in protein expression.

### 3.3 Integrated analysis of DEPs and DMs

An integrated analysis of the proteomic and metabolomic data provided a comprehensive overview of the coordinated cellular response to MB-PDT (Table 1). The combined datasets revealed a multi-faceted disruption of key biological processes.

**Table 1:** Representative molecules co-disrupted by MB-PDT, categorized by key biological processes.

Biological process/pathway	Representative proteins (DEPs)	Representative metabolites (DMs)
Ribosome biogenesis & translation	Multiple 40S & 60S ribosomal proteins (↓) Structural constituent of ribosome (↓)	—
Amino acid & peptide metabolism	Enzymes in alanine, aspartate, glutamate, arginine metabolism pathways (↓)	Multiple dipeptides (e.g., Asp-Val, Ala-Leu, Ile-Val) (↑) N-Acetyl-L-leucine (↑)
Redox homeostasis & oxidative stress response	Glutathione S-transferase (GST) (↓) Alternative oxidase (AOX) (↓) aldehyde dehydrogenase (↓) glyoxalase family protein (↓)	Cystine (↑) Val-Met (↑) Hgln (↓)
Cell membrane integrity & transport	C4-methylsterol oxidase (↓) ABC transporter (↓) Acyl-CoA desaturase (↓) K(+)/H(+) antiporter 1 (↓) member of major facilitator superfamily (↓)	Butyrylcarnitine (↑) O-propanoylcarnitine (↑) putrescine (↑) methylene blue (↓)
Energy & carbon metabolism	Enzymes in glycolysis/gluconeogenesis pathway (↓)	Kanosamine (↓)
Virulence factor expression	Subtilisin-like protease 7 (↓) phospholipase A2 (↓) chitinase (multiple forms) (↓) hemolysin-III family protein (↓) small secreted protein (↓)	—

Arrows indicate the direction of change observed in the MB-PDT group compared to the control group. ↑: upregulation or increased abundance; ↓: downregulation or decreased abundance.

In ribosome biogenesis & translation, an extensive downregulation was observed of multiple 40S and 60S ribosomal proteins. Concurrently, in amino acid & peptide metabolism, enzymes in key metabolic pathways were downregulated, while multiple dipeptides (e.g., Asp-Val, Ala-Leu, Ile-Val) and N-Acetyl-L-leucine accumulated. For cell membrane integrity & transport, the downregulation of proteins such as C4-methylsterol oxidase and an ABC transporter was accompanied by altered levels of metabolites like butyrylcarnitine (upregulated) and methylene blue (downregulated).

Furthermore, the downregulation of antioxidant enzymes was observed, including glutathione S-transferase (GST) and alternative oxidase (AOX), indicating alterations in redox homeostasis. Finally, we recorded a suppression of enzymes in energy & carbon metabolism and a significant downregulation of key virulence factor proteins, including subtilisin-like protease 7 and phospholipase A2. In summary, the integrated analysis reveals a coordinated downregulation of proteins involved in core cellular functions, alongside specific and significant alterations in the corresponding metabolite pools.

## 4 Discussion

By integrating DIA proteomics with untargeted metabolomics, this study provides, to our knowledge, the first system-level characterization of the molecular response of clinical *Microsporum canis* isolates to MB-PDT. This approach allowed us to elucidate the multifaceted fungicidal action of MB-PDT, revealing a complex network of molecular perturbations rather than a single mode of action. The core mechanisms identified include the disruption of cell membrane homeostasis, the suppression of key virulence-associated proteins, the impairment of the antioxidant defense system, and a profound disruption of core biosynthetic processes.

### 4.1 MB-PDT disrupts cell membrane homeostasis

Our integrated omics data support the hypothesis that the cell membrane system is a primary site of MB-PDT-induced damage. The significant downregulation of C4-methylsterol oxidase, a key enzyme in the ergosterol biosynthesis pathway, suggests that MB-PDT impairs the production of the membrane’s principal sterol. This effect is likely attributable to ROS-mediated lipid peroxidation, which reduces ergosterol content and compromises membrane integrity (da Silva et al., 2025). This finding aligns with previous PDT studies in other pathogenic fungi that also identified the cell membrane as a primary target, often involving oxidative damage to cellular structures and increased membrane permeability (Dai et al., 2012; Wu and Hu, 2022). Concurrently, the reduced abundance of ABC transporters may compromise the cell’s ability to efflux xenobiotics, potentially leading to higher intracellular photosensitizer concentrations and amplified photodamage. This is consistent with findings that PDT interferes with efflux gene expression in resistant fungal biofilms (Štefánek et al., 2022). The downregulation of K(+)/H(+) antiporter 1 further indicates a disruption of transmembrane ion gradients, which are critical for cellular energy and pH homeostasis, similar to observations of potassium ion efflux and loss of plasma membrane barrier properties in PDT-treated yeast cells (Dai et al., 2012). Metabolomic data corroborate these findings, as the accumulation of butyrylcarnitine and O-propanoylcarnitine indicates impaired mitochondrial beta-oxidation, a process reliant on mitochondrial membrane integrity. Such acylcarnitine elevations are characteristic of beta-oxidation defects, where enzymatic blockages lead to a rise in C4:0 (butyrylcarnitine) and related species, causing lipotoxicity and oxidative stress (Guerra et al., 2022). Collectively, these data suggest that MB-PDT destabilizes membrane homeostasis through a coordinated disruption of membrane synthesis, transport functions, and associated energetic processes. This conclusion is consistent with reviews highlighting the multi-target oxidative effects of MB-PDT in cutaneous fungal infections (Cardozo et al., 2024).

### 4.2 Suppression of secreted virulence factors attenuates pathogenic potential

Beyond its direct cytotoxicity, the data suggest that MB-PDT significantly attenuates the pathogenic potential of *M. canis* by suppressing the expression of key secreted enzymes. The marked downregulation of subtilisin-like protease 7 is particularly significant, as this keratinase is essential for degrading host tissues during infection. This finding is analogous to the role of *Sub3* in related fungi, where its inhibition was shown to reduce fungal attachment to feline corneocytes without impacting invasion (Vite-Garín et al., 2024). The diminished abundance of phospholipase A2 and multiple chitinases further suggests a reduced capacity for host cell disruption and cell wall remodeling. For example, the deletion of *BbPLA2* in *Beauveria bassiana* impairs lipid droplet homeostasis and host immune repression, leading to a 40% virulence reduction in *Galleria mellonella* models (Deng et al., 2022). Similarly, silencing a chitinase gene in *Puccinia striiformis* decreases uredinia formation while elevating host H₂O₂ accumulation (Guo et al., 2023; Valdez et al., 2023). The simultaneous suppression of these diverse virulence factors indicates that MB-PDT exerts a broad-spectrum inhibitory effect. Crucially, this implies that MB-PDT does not merely kill the fungus but also “disarms” any surviving cells by stripping them of essential invasion tools. Consequently, these functionally impaired survivors would have a significantly reduced capacity to re-establish infection, thereby minimizing the risk of relapse. This hypothesis is supported by analogous studies in *Candida* species, where aPDT was shown to compromise the physiological fitness of surviving cells, rendering them more susceptible to host defenses and subsequent treatment (da Silva et al., 2025; Du et al., 2024).

### 4.3 Dual impairment of the fungal antioxidant defense system

Current findings suggest that MB-PDT impairs the fungal redox system through a dual mechanism: inducing massive oxidative stress while simultaneously suppressing key antioxidant defenses (Wu and Hu, 2022). The downregulation of cytoplasmic enzymes such as GST and aldehyde dehydrogenase indicates a diminished capacity to detoxify ROS-induced secondary products like lipid peroxides and reactive aldehydes (Wangsanut and Pongpom, 2022; Tang et al., 2023). This proteomic observation is supported by the metabolomic detection of accumulating L-cystine, a biomarker of a depleted glutathione pool and heightened oxidative stress (He et al., 2023). Concurrently, the suppression of mitochondrial AOX suggests the impairment of a critical pathway that mitigates mitochondrial ROS production under stress. Experiments with AOX mutants in *Candida albicans* showed that the absence of AOX led to elevated mitochondrial ROS levels and growth defects under high-iron conditions (Sharma et al., 2023). This concurrent impairment of defense mechanisms in both the cytoplasm and mitochondria likely creates a profound state of oxidative distress, contributing to the widespread molecular damage observed (Wangsanut et al., 2023; Park and Son, 2024).

### 4.4 Profound disruption of core biosynthetic machinery

The culminating effect of MB-PDT is the systemic failure of the cell’s core biosynthetic machinery. Functional enrichment analysis identified “Ribosome” as the most significantly affected pathway, a finding substantiated by the extensive downregulation of proteins from both the 40S and 60S ribosomal subunits. This widespread suppression likely stems from two complex mechanisms. First, ribosomes are nucleoprotein complexes that are highly susceptible to oxidative damage; the singlet oxygen and free radicals generated by MB-PDT can directly oxidize ribosomal RNA and proteins, leading to chemical modifications, strand cleavage, and destabilization (Tanaka and Chock, 2021; Zinskie et al., 2018). Such oxidative lesions impair ribosomal function and trigger degradation pathways (Yang et al., 2023). Second, the arrest of protein synthesis represents a critical adaptive response to lethal stress. Translation is one of the most energy-consuming cellular processes. By shutting down ribosome biogenesis and translation, the fungus attempts to conserve ATP for essential repair mechanisms and survival, while simultaneously preventing the production of error-prone or misfolded proteins in an oxidative environment (Tanaka and Chock, 2021).

This collapse of the translational machinery mirrors findings in previous studies on other pathogenic fungi. For instance, in *Trichosporon asahii* biofilms exposed to ALA-PDT, downregulation of genes involved in amino acid metabolism essential for protein synthesis was observed, further disrupting translational processes (Luo et al., 2025). This consistency across studies suggests that the dismantling of protein synthesis is not merely a strain-specific response of *M. canis*, but a conserved, fundamental mode of action by which PDT induces cell death in fungal pathogens (Yang et al., 2023). This widespread inhibition of the cell’s synthetic and metabolic core provides a compelling molecular explanation for the potent fungicidal activity of MB-PDT (Wu and Hu, 2022; Ziental et al., 2021).

### 4.5 Clinical implications, strengths, and limitations

The multi-target mechanism of MB-PDT elucidated here positions it as a promising therapeutic strategy, particularly in the context of rising antifungal resistance (Sacheli and Hayette, 2021). Conventional agents, such as terbinafine, exert high selective pressure on a single enzymatic target (squalene epoxidase). This pressure facilitates the evolution of resistance through single-gene mutations, such as L393F and F397L substitutions leading to MICs >4 μg/mL (Sacheli and Hayette, 2021). In contrast, MB-PDT inflicts widespread oxidative damage across a broad range of cellular components, including C4-methylsterol oxidase, ABC transporters, subtilisin-like protease 7, and dozens of ribosomal proteins. This damage is mediated via ROS generation that causes cell wall perforation and organelle disruption, as shown in *in vitro* studies on resistant fungi (da Silva et al., 2025; Zheng et al., 2023). The genetic and physiological hurdles for developing resistance to such a multifaceted attack are substantially higher. This resilience is supported by clinical evidence showing that PDT can achieve >80% clinical clearance in resistant cases without inducing resistance (Thakur et al., 2025). This inherent resilience against resistance development, coupled with its low cost and the established safety profile of topical methylene blue at doses below 2 mg/kg (Seitkazina et al., 2022; Bistas and Sanghavi, 2023), underscores the therapeutic potential of MB-PDT as a durable alternative to conventional therapies.

This study’s strengths lie in its integrated omics approach, which offers an unbiased, system-level view of the fungal response, and its use of clinical isolates, which enhances translational relevance. The detailed mechanistic map provided in this study also substantiates the low resistance potential of MB-PDT. Nevertheless, certain limitations must be acknowledged. This *in vitro* model does not fully recapitulate the complex host-pathogen interactions of an *in vivo* setting. The analysis was also limited to a single time point and photosensitizer, and the correlational nature of omics data necessitates future functional studies to establish causality. Notably, the absence of a conventional antifungal control group precludes a direct comparison of affected pathways. Future research should therefore aim to validate these findings in animal models, perform time-course and dose–response analyses, and directly compare the molecular impact of MB-PDT with that of standard antifungals on both susceptible and resistant strains. Furthermore, to gain a deeper understanding of the cell’s oxidative state, future studies should employ redox proteomics. Unlike standard proteomics which measures protein abundance, redox proteomics can identify and quantify specific oxidative modifications on proteins. This approach will be instrumental in distinguishing between proteins that are downregulated and those that are functionally inactivated by ROS, thereby pinpointing the direct molecular targets of MB-PDT. A critical translational challenge remains the delivery of sufficient light through physical barriers like the nail plate. Therefore, developing innovative light-delivery systems is imperative to advance this promising modality into clinical practice.

## 5 Conclusion

**Figure 6:**
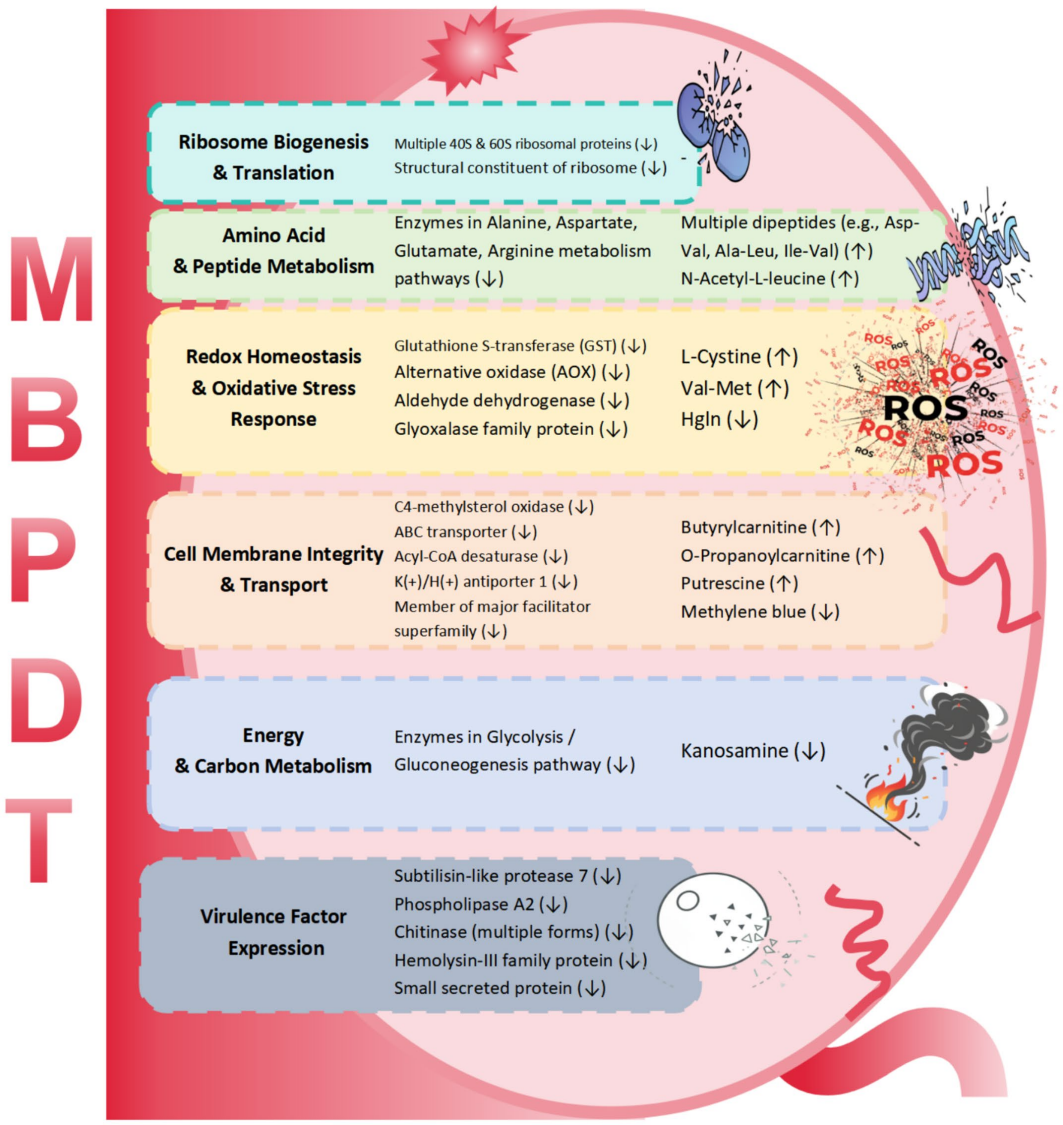
A schematic model summarizing the multi-pronged fungicidal mechanism of methylene blue-mediated photodynamic therapy (MB-PDT) against *M. canis*. MB-PDT induces massive oxidative stress (ROS), which simultaneously disrupts cell membrane integrity, suppresses key virulence factors, and impairs the antioxidant defense system. These effects lead to a systemic collapse of core biosynthetic processes, including ribosome biogenesis, protein translation, and energy metabolism, ultimately causing fungal cell death.

By integrating proteomics and metabolomics, this study reveals the multi-pronged fungicidal mechanism of MB-PDT against *M. canis*. The findings indicate that MB-PDT simultaneously disrupts cell membrane integrity, downregulates key virulence factors, and suppresses core biosynthetic pathways, leading to a systemic collapse of cellular homeostasis (Figure 6). This multi-faceted impact provides a strong molecular rationale for MB-PDT as a durable therapeutic strategy, presenting a significant advantage over single-target antifungals that are prone to resistance. While these *in vitro* findings are compelling, they highlight the need for future research. Specifically, further studies are necessary to distinguish the primary molecular targets of oxidative damage from secondary metabolic consequences, and functional validation of the identified molecular changes (e.g., via gene silencing) and evaluation in *in vivo* animal models are essential next steps. These investigations will be crucial for confirming these mechanisms and translating our findings into optimized clinical protocols to combat dermatophytosis.

## Data Availability

The datasets presented in this study can be found in online repositories. The names of the repository/repositories and accession number(s) can be found in the article/supplementary material.
